# Effect of chitosan/inorganic nanomaterial scaffolds on bone regeneration and related influencing factors in animal models: A systematic review

**DOI:** 10.3389/fbioe.2022.986212

**Published:** 2022-10-26

**Authors:** Anjie Guo, Yi Zheng, Yu Zhong, Shuixue Mo, Shanbao Fang

**Affiliations:** College of Stomatology, Guangxi Medical University, Nanning, China

**Keywords:** chitosan, inorganic nanomaterials, bone regeneration, animal models, calvarial bone defects

## Abstract

Bone tissue engineering (BTE) provides a promising alternative for transplanting. Due to biocompatibility and biodegradability, chitosan-based scaffolds have been extensively studied. In recent years, many inorganic nanomaterials have been utilized to modify the performance of chitosan-based materials. In order to ascertain the impact of chitosan/inorganic nanomaterial scaffolds on bone regeneration and related key factors, this study presents a systematic comparison of various scaffolds in the calvarial critical-sized defect (CSD) model. A total of four electronic databases were searched without publication date or language restrictions up to April 2022. The Animal Research Reporting of *In Vivo* Experiments 2.0 guidelines (ARRIVE 2.0) were used to assess the quality of the included studies. Moreover, the risk of bias (RoB) was evaluated *via* the Systematic Review Center for Laboratory Animal Experimentation (SYRCLE) tool. After the screening, 22 studies were selected. None of these studies achieved high quality or had a low RoB. In the available studies, scaffolds reconstructed bone defects in radically different extensions. Several significant factors were identified, including baseline characteristics, physicochemical properties of scaffolds, surgery details, and scanning or reconstruction parameters of micro-computed tomography (micro-CT). Further studies should focus on not only improving the osteogenic performance of the scaffolds but also increasing the credibility of studies through rigorous experimental design and normative reports.

## Introduction

Alveolar bone dehiscence or fenestration, which may cause gingival recession, is one of the common sequelae of orthodontic treatment. The loss of periodontal support tissues compromises the impacted teeth both esthetically and healthily. Thus, bone augmentation may be required for those who have had alveolar dehiscence or fenestration before orthodontics or are assessed as having a high risk of morbidity. This bone transplantation surgery is generally carried out using auto- or xenogenic bone grafts, with satisfactory effects. However, the most substantial disadvantages of these materials are their constrained resources, additional trauma, and high costs. It is indispensable to conduct research and development for bone substitute materials that combine ideal efficacy and affordability.

Recently, bone tissue engineering (BTE) has undergone rapid progress, providing a novel approach for bone regeneration. By simulating the morphology and function of the native skeletal, bone substitute materials would be constructed, composed of scaffold and growth factors that confer osteoconductivity and osteoinductivity to these materials. Numerous materials have been revealed to be suitable for BTE. Natural organic macromolecules such as chitosan (CS), collagen, silk fibroin (SF), and hyaluronic acid have been broadly studied for their biocompatibility and biodegradability (Jin et al., 2021; Sun et al., 2021), while inorganic minerals like hydroxyapatite, calcium phosphates, calcium silicates, bioglass, and bioceramics are also extensively applied due to their bioactivity and osteoconductivity (Cheah et al., 2021; Kamboj et al., 2021). Even though each type of material has advantages of its own, it is still challenging to meet all of the requirements of BTE. For instance, scaffolds would have poor mechanical properties when organic macromolecules are individually used or a slow degradation velocity when only inorganic minerals are employed. Accordingly, constructing scaffolds with hybrid materials becomes a promising tissue engineering strategy, combining the advantages of both organic/inorganic materials (Jayash et al., 2021).

As a natural biomacromolecule, CS has been utilized as the matrix of scaffolds owing to its superior biocompatibility, biodegradability, antibiosis, and properties of adhesion and adsorption (Guo et al., 2021). Nonetheless, as a result of its inherent drawback of poor mechanical properties (Sukpaita et al., 2021), combining CS with various inorganic materials for modification of scaffolds has become a hotspot of research (Liu et al., 2021). Compared with macro- or microscale materials, nanomaterials are nano-sized, ranging from 1–100 nm in at least one dimension (Rajula et al., 2021). Due to the small particle size and high surface area, their macroscopic properties are strongly affected by the interaction between atoms and molecules at the nanoscale, such as mechanical strength, cell adhesive property, and the ability to mimic the hierarchical structure of the extracellular matrix (Dvir et al., 2011; Pina et al., 2015; Abdollahiyan et al., 2021).

In the development of biomaterials, *in vivo* animal experiments are imperative for translating *in vitro* results to the clinic. Therefore, to better evaluate the performance of composite scaffolds and obtain data with optimal relevance to the clinical situation, it is crucial to select an appropriate animal model for mimicking the *in vivo* environment where biomaterials play a role (Muschler et al., 2010). As for the species employed in BTE research, murines, rabbits, canines, and pigs are commonly used, while murines are used most extensively considering their lower costs and convenient operation process (Gomes and Fernandes, 2011). To the preparation methods of modeling in BTE, calvarial bone or extremity bone is the most common location to create round or segmental critical-size defects, respectively (Vajgel et al., 2014; Sparks et al., 2020). Taking into account that the skull and the alveolar bone have the same developmental mode, i.e., intramembranous ossification, belong to the flat bone, and have similar force, the calvaria can better ape the environment of the alveolar bone than the long bone. Moreover, researchers have reached a consensus on the application of the calvaria CSD model and standardized the preparation process, which makes the model reproducible and reliable (McGovern et al., 2018).

After constructing the animal model, it is crucial to evaluate bone regeneration. Histomorphometry has been applied for many years to assess bone parameters at the cellular level, but it is a destructive and 2D technique (Lyu and Lee, 2021). With high resolution, micro-CT provides 3D examinations of specimens, offering details about the volume and morphology of bones, which can determine the bone quality by variables such as bone volume fraction, trabecular number, trabecular thickness, and bone mineral density (Bouxsein et al., 2010; Akhter and Recker, 2021).

At present, although numerous investigations related to biomaterials have been carried out, little information is available about the quality of these studies, and few composite scaffolds have been translated into clinical application. Based on this context, this study aims to conduct a systematic comparison, focusing on the bone regeneration effects of various CS/inorganic nanomaterial composite scaffolds on calvarial bone defect models, and to identify the critical factors influencing the osteogenic properties of scaffolds *in vivo*. In addition, in order to guide future studies, quality assessment and evaluation of the risk of bias are accomplished for previous studies.

## Methods

### Protocol, registration, and search strategy

This systematic review has not been registered. The protocol and this report follow the PRISMA guidelines.

Our research questions were turned into the PICO (Participant, Intervention, Comparison, and Outcome) model to formulate the search strategy. The literature search was conducted at PubMed, Web of Science, Embase, and Cochrane Library up to April 2022 for publications. No restrictions on language in the literature search were reported. The detailed search terms are given in Table 1.

**TABLE 1:** Search strategies in different databases.

Database	Search strategy
PubMed and Cochrane library	#1. rat OR rodent OR pig OR dog OR monkey OR rabbit OR mice OR murine OR animals OR animals[MeSH]
	#2. (chitosan OR poliglusam OR chitosan[MeSh]) AND (nano* OR “nanostructures” [MeSH])
	#3. (bone regenerate*) OR (bone format*) OR osteogen* OR (bone heal*) OR (bone repair*) OR (bone transplantation) OR (bone reconstruct*) OR ossificat* OR (bone augment*) OR (bone tissue engineering) OR “bone transplantation”[MeSH] OR “bone regeneration”[MeSH] OR “bone transplantation”[MeSH]
	#4. #1 AND #2 AND #3
Embase	#1. ‘rat’/exp OR rat OR ‘rodent’/exp OR rodent OR ‘pig’/exp OR pig OR ‘dog’/exp OR dog OR ‘monkey’/exp OR monkey OR ‘rabbit’/exp OR rabbit OR ‘mice’/exp OR mice OR ‘murine’/exp OR murine OR ‘animals’/exp OR animals OR ‘animal’/exp OR animal
	#2. (‘chitosan’/exp OR chitosan OR poliglusam) AND (‘nanomaterial’/exp OR nano*)
	#3. ‘bone regeneration’/exp OR ‘bone regenerat*’; OR ‘bone format*’ OR osteogen* OR ‘bone heal*’ OR ‘bone repair*’ OR ‘bone remodeling’/exp OR ‘bone transplantation’ OR ‘bone transplantation’/exp OR ‘bone reconstruct*’ OR ‘ossification’/exp OR ossificat* OR ‘bone augment*’ OR ‘bone tissue engineering’
	#4. #1 AND #2 AND #3
WOS	#1. rat OR rodent OR pig OR dog OR monkey OR rabbit OR mice OR murine OR animal*
	#2. (chitosan OR poliglusam) AND nano*
	#3. “bone regenerate*” OR “bone format*” OR osteogen* OR “bone heal*” OR “bone repair*” OR “bone transplantation” OR “bone reconstruct*” OR ossificat* OR “bone augment*” OR “bone tissue engineering”
	#4. #1 AND #2 AND #3

### Inclusion/exclusion criteria

Two reviewers will independently carry out the selection of studies and make decisions about eligibility. If the relevance of a study report is unclear, we will review the full text and resolve all disagreements by discussion.

The inclusion criteria were as follows:

1) *In situ* bone regeneration research of CS or its derivatives/inorganic nanomaterial composite scaffolds, with at least one experimental group free of substances or cells that can promote osteogenesis.
2) The animal model was the calvaria defect model.
3) Quantitative evaluation was accomplished through micro-CT, and the outcome measures contained at least BV/TV, BMD, Tb.Th, or Tb.N.

The exclusion criteria were as follows:

1) CS or nanomaterials served as the coating of scaffolds rather than the components of the matrix.
2) Except for skull defects, animals had metabolic bone disease, infection, tumor, or immunodeficiency simultaneously.
3) Discrepancies in outcome measures existed between figures and literal descriptions, affecting data accuracy.

### Outcome measure

**FIGURE 1:**
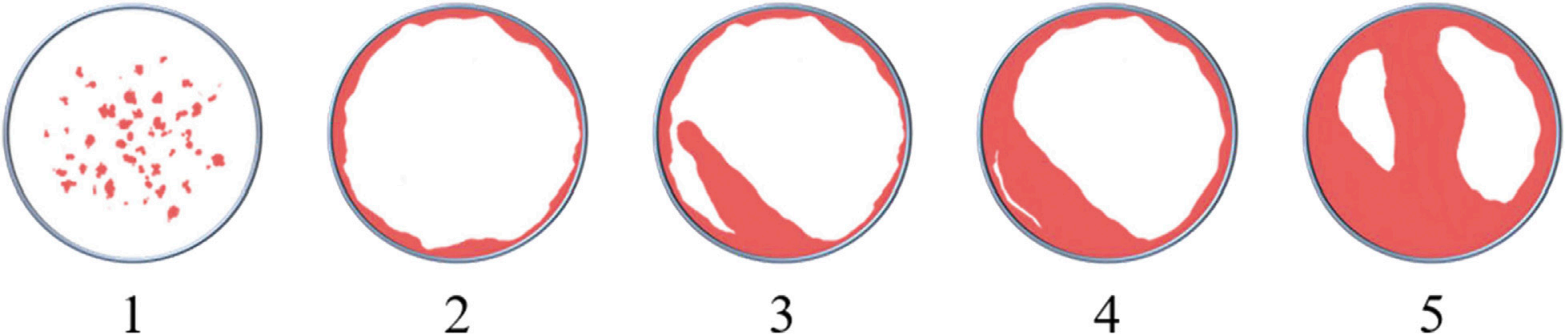
Schematic diagram of the scoring system.

The primary outcome measures were the results of the quantitative analysis of reconstruction data obtained from micro-CT scanning as follows:

1) Bone volume/total volume (BV/TV);
2) Bone mineral density (BMD);
3) Trabecular thickness (Tb.Th);
4) Trabecular number (Tb.N);
5) Scoring of reconstruction images: a semi-quantitative assessment of images reconstructed from micro-CT data was conducted to interpret the osteoconductivity of scaffolds. The scoring system (see Table 2 and Figure 1) was modified from that of Young et al. (2009) ‘s.

**TABLE 2:** Scoring system of images derived from the reconstruction.

Description	Score
No bone formation in the defect area	0
Punctate or needle-shaped mineralization tissue scattered throughout the defect	1
Tissue mineralizing only along defect edge	2
Bony bridge forming but not connected to sides of the defect	3
Bony bridging over the partial length of the defect	4
Bony bridging over the diameter of the defect	5
Bone covered the whole defect area	6

### Data extraction

Data extraction was performed with a predefined form, and Microsoft Excel (Microsoft Office 2016; Microsoft, Redmond, WA, United States) was utilized to record data. More specifically, only groups implanted with cell-free, growth factor-free, and medicine-free scaffolds could be recorded. The extracted data include five main entries.

1) Study details: author(s) and publication year.
2) Characteristics of animals: species, strain, age, weight, gender, and total number.
3) Interventions: defect location and diameter and healing period.
4) Details of the implanted scaffolds: characteristics of CS and nanomaterials, preparation methods, and properties of the scaffolds.
5) Outcome measures: BV/TV, BMD, Tb.Th, and Tb.N are calculated from micro-CT and related parameters.

Additionally, when specific data were unavailable in the original articles, data were extracted from the coordinate diagrams *via* Engauge Digitizer software (http://digitizer.sourceforge.net/).

### Quality assessment

The quality of each included study was assessed according to the ARRIVE 2.0 guidelines (Percie du Sert et al., 2020). The ARRIVE 2.0 guidelines contain 21 main items: 1. study design; 2. sample size; 3. inclusion and exclusion criteria; 4. randomization; 5. blinding; 6. outcome measures; 7. statistical methods; 8. experimental animals; 9. experimental procedures; 10. results; 11. abstract; 12. background; 13. objectives; 14. ethical statement; 15. housing and husbandry; 16. animal care and monitoring; 17. interpretation/scientific implications; 18. translation; 19. protocol registration; 20. data access; and 21. declaration of interests. According to the authors, the first 10 items belong to the essential set that must be included in the study, or the reliability of the research cannot be assessed. The remaining 11 items are included in the recommended set, which serves as complements for the essential set.

Each main item, which consisted of several sub-items, was scored, being marked as “reported (= 2 points)” if the article complied with all sub-items, “not reported (= 0 points)” if it did not, and “not clear (= 1 point)” if only part of the sub-items were provided. After scoring, for evaluating the quality of each study, a predefined quality coefficient was calculated as the sum of all the 21 items and divided by 42. Depending on the coefficient, the quality of the studies was classified as “excellent (0.8–1),” “average (0.5—0.8),” and “poor (<0.5)” (García-González et al., 2021).

### Evaluation of the risk of bias

SYRCLE’s RoB tool was employed to evaluate the risk of bias in each included study (Hooijmans et al., 2014). The types of bias in this tool include selection, performance, detection, attrition, reporting, and other sources of bias not covered in the tool. More specifically, these were translated into 10 items, requesting the evaluators to answer questions *via* “Yes,” “No,” or “Unclear” (see Table 3). Each item was assessed as a low RoB if the answers of all the sub-items were “Yes,” high RoB if at least one answer of a sub-item was “No,” and unclear RoB for the rest situation. For each study, the overall RoB was assessed as low if all the items were “Yes,” high if at least one item was “No,” and “Unclear” for other situations.

**TABLE 3:** SYRCLE’s RoB tool for the evaluation of risk bias of studies. A total of 10 sources of bias are translated into 10 questions, requesting the evaluators to answer with “Yes,” “No,” or “Unclear.” Each item is assessed as a low RoB if the answers of all the sub-items were “Yes,” high RoB if at least one answer of a sub-item is “No,” and unclear RoB for the rest of the situations.

Item	Type of bias	Domain	Review author judgment
1	Selection bias	Sequence generation	Was the allocation sequence adequately generated and applied?
2	Selection bias	Baseline characteristics	Were the groups similar at baseline, or were they adjusted for confounders in the analysis?
3	Selection bias	Allocation concealment	Was the allocation adequately concealed?
4	Performance bias	Random housing	Were the animals randomly housed during the experiment?
5	Performance bias	Blinding	Were the caregivers and/or investigators blinded from knowledge of which intervention each animal received during the experiment?
6	Detection bias	Random outcome assessment	Were animals selected at random for outcome assessment?
7	Detection bias	Blinding	Was the outcome assessor blinded?
8	Attrition bias	Incomplete outcome data	Were incomplete outcome data adequately addressed?
9	Reporting bias	Selective outcome reporting	Are reports of the study free of selective outcome reporting?
10	Other	Other sources of bias	Was the study apparently free of other problems that could result in a high risk of bias?

### Data processing and statistical analysis

For controlling the variable, the mean BV/TV (mBV/TV) was obtained by dividing the BV/TV by the healing period. The mean BMD (mBMD) was calculated in the same way.

Statistical data were shown as mean ± SD. If the data in the original article were shown as the median and interquartile range (IQR), they were transformed into mean ± SD *via* an online calculator (www.math.hkbu.edu.hk/∼tongt/papers/median2mean.html) (Wan et al., 2014; Luo et al., 2018; Shi et al., 2020).

## Results

### Search and screening results

**FIGURE 2:**
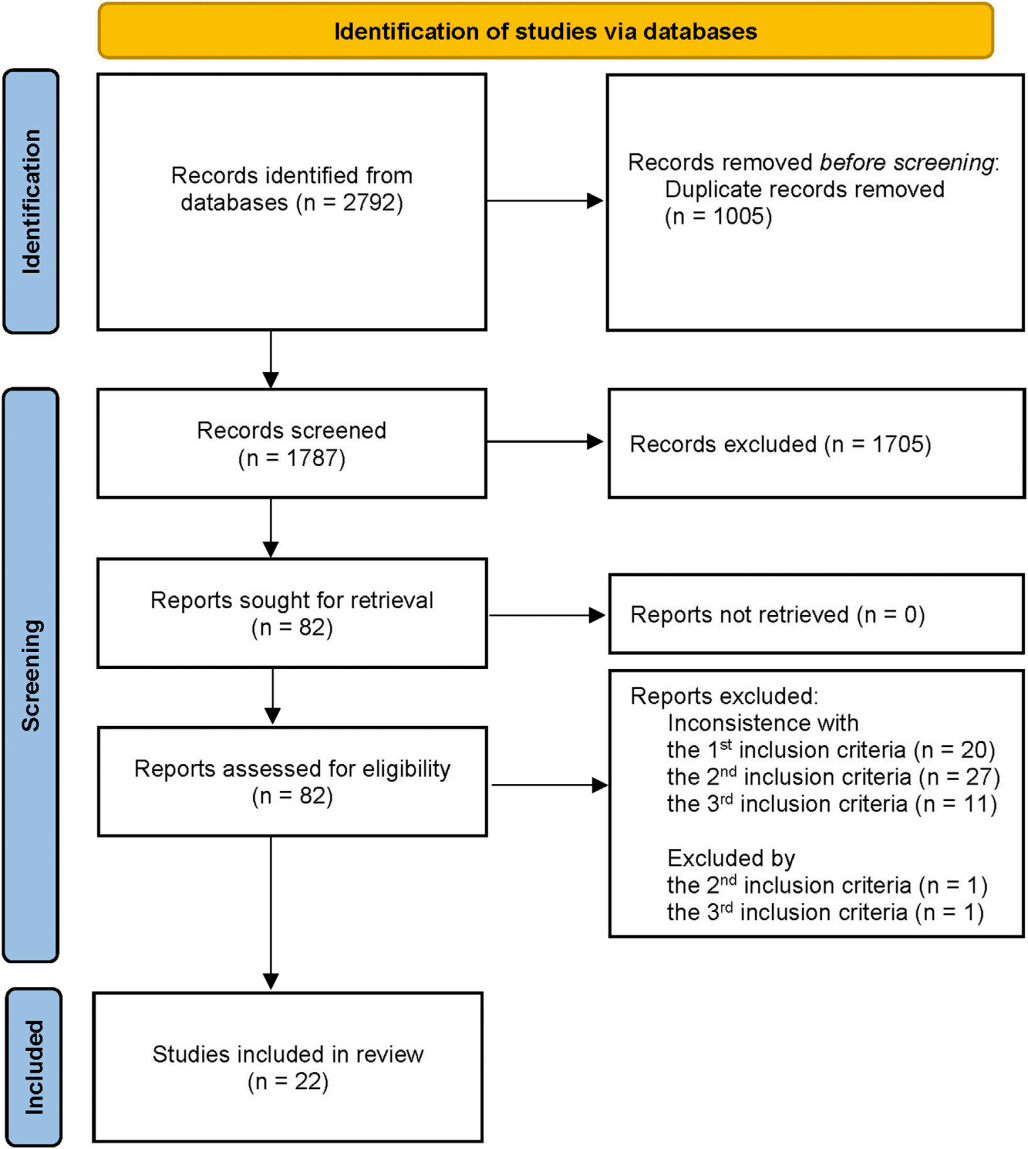
Flow chart of studies selected for the systematic review.

A total of 2,792 literature studies were acquired from those four databases, and 82 were selected and read as full-text after screening the titles and abstracts. Finally, 22 studies met our eligibility criteria and were included in this study for further qualitative analysis (Figure 2).

### Characteristics of study subjects

All the included studies applied murines to construct the calvaria CSD model since their calvarial bones with the proper size were convenient to process. Rats served as subjects in 95% (21 of 22) of the studies, with Sprague–Dawley (SD) rats in most of the research and Wistar rats in one study. Male rats were applied in 14 of them and female rats in four, while the remaining three literature studies did not report the gender of the animals (Calis et al., 2017; Hu et al., 2018; Zhao P. P. et al., 2019). Moreover, 13 studies illustrated the age of rats, ranging from 8–12 weeks in most of them, while juvenile rats (Sengupta, 2013) of no more than 8 weeks were used in one research (Ji et al., 2017). Animal body weight and sample size have not been illustrated in a few studies. The results are shown in Table 4.

**TABLE 4:** Baseline characteristics of study subjects. SD: Sprague–Dawley; W: weeks; M: male.

Author (Year)	Animal	Strain	Age	Weight	Gender	Total number
M. Calis et al. (2017)	Rat	SD	—	200–250 g	—	30
X. Ding et al. (2019a)	Rat	SD	8W	200–250 g	M	24
Y. Ji et al. (2017)	Rat	SD	6W	120 ± 15 g	M	40
D. Zhou et al. (2017)	Rat	SD	12W	250–300 g	M	30
Lee et al. (2020)	Mouse	CD-1	8–10W	—	M	12
F. Liao et al. (2019)	Rat	SD	—	—	M	20
Y. P. Guo et al. (2015)	Rat	SD	12W	250–300 g	M	18
T. W. Sun et al. (2017)	Rat	SD	8W	250–300 g	M	24
X. Y. Peng et al. (2019)	Rat	SD	—	300–350 g	M	20
H. Hu et al. (2018)	Rat	SD	8W	250 ± 25 g	—	20
Y. Zhao et al. (2019a)	Rat	SD	—	300 g	M	—
Y. X. Chen et al. (2017)	Rat	SD	12W	300–350 g	M	20
Y. Li et al. (2018a)	Rat	Wistar	12W	330 ± 19 g	M	20
T. Wu et al. (2020a)	Rat	SD	12W	—	M	24
J. Wu et al. (2019)	Rat	SD	14W	300–350 g	M	30
Chen et al. (2022)	Rat	SD	—	150 g	F	36
Zhao et al. (2019b)	Rat	SD	8W	250 ± 25 g	—	20
Tang et al. (2020)	Rat	SD	—	250–300 g	M	30
Hu et al. (2019)	Rat	SD	12W	—	F	20
Chen et al. (2018)	Rat	SD	8W	—	M	20
Chen et al. (2021a)	Rat	SD	—	200 g	F	24
Yu et al. (2021)	Rat	SD	—	200–250 g	F	15

### Interventions

#### Characteristics of scaffolds

As shown in Table 5, the properties of chitosan utilized in different studies were distinguished. The degree of deacetylation (DD) of chitosan was reported in nine studies, most of which was above 80%. Only one study used chitosan with a low DD, more specifically, 60% (Li Y. et al., 2018). Except for the DD, the molecular weight (MW) also plays an important role in the performance of chitosan. Only three described the MW of chitosan directly, with the orders of magnitude varying from 105–106 (Guo et al., 2015; Liao et al., 2019; Wu et al., 2019). In addition, Lee et al. (2020), Chen et al. (2022), Chen Y. Q. et al. (2021), and Wu T. et al. (2020) modified the sidechain of chitosan by grafting different functional groups and harvested chitosan derivatives for improving physicochemical properties, such as solubility and cross-linked degree.

**TABLE 5:** Characteristics of scaffolds. MW: molecular weight; GP: glycerophosphate; DEX: dextran; Col: collagen; PGCS: phytochemical-grafted chitosan; LAP: laponite; MCS: mesoporous calcium silicate; MS: magnesium silicate; LDH: layered double hydroxide; QCS: NC-CL: non-covalent cross-linking; C-CL: covalent cross-linking; FD: freeze–drying; BG: bioactive glass; SF: silk fibroin; PMCS: phosphate modified methacryloyl chitosan; CnHA: carbonated nHA; MnHA: mesoporous nHA.

Author (year)	Morphology of the scaffold	Nature of chitosan	Nanomaterial	Nano-filler proportion	Other material	Preparation	Pore size	Degradation rate (medium)
M. Calis et al. (2017)	Gel	High viscosity, DD = 80%	B-HA	—	GP	NC-CL	—	—
X. Ding et al. (2019b)	Gel	—	Sr-HA	6%	DEX	C-CL	100–300 μm	20% at 4 W (37°C PBS)
Y. Ji et al. (2017)	Porous	—	Col-HA	22%	PLGA	FD, −20°C	—	12.86% at 4 W (37°C PBS)
D. Zhou et al. (2017)	Porous	—	WH	40%	—	FD	70–150 μm	Ion release experiments
Lee et al. (2020)	Gel	PGCS	LAP	1%	—	C-CL	—	40%–50% at 6 W (37°C PBS)
F. Liao et al. (2019)	Porous	MW = ∼10^5^, DD = 90%	Gd-MCS	50%	—	FD, −20 °C	∼200 μm	—
Y. P. Guo et al. (2015)	Porous	MW = 4×10^5^, DD = 85%	HA	—	—	needle-punching	30–100 μm	—
T. W. Sun et al. (2017)	Porous	Medium viscosity	MS-HA	70%	—	FD, −20°C	200–300 μm	Ion release experiments
X. Y. Peng et al. (2019)	Porous	DD = 90%	La-MCS	50%	—	FD, −20°C	∼200 μm	13.41% at 1 W (37°C water)
H. Hu et al. (2018)	Porous	—	LaPO_4_	50%	—	FD, −20°C	∼200 μm	—
Y. Zhao et al. (2019b)	Porous	—	Fe-HA	—	COL	FD	100–300 μm	3.15% at 1 W (37°C PBS with lysozyme)
Y. X. Chen et al. (2017)	Porous	—	MgAl-LDH	40%	—	FD	∼100 μm	—
Y. Li et al. (2018b)	Porous	DD ≥ 60%	HA	66%	PLGA	FD, −80°C	∼100 μm	—
T. Wu et al. (2020b)	Porous	low MW, DD = 80%CS	Sr-HA	0.28%	—	FD, −80°C	—	—
J. Wu et al. (2019)	Gel	MW = 2.2×10^6^ DD = 94%	Cu-BG	1%	GP/SF	NC-CL	50–100 μm	—
Chen et al. (2022)	Gel	PMCS	MgO	1%	—	NC-CL	50–100 μm	20% at 4 W (37 °C PBS)
Zhao et al. (2019c)	Porous	—	GdPO_4_	50%	—	FD, −60 °C	∼200 μm	—
Tang et al. (2020)	Porous	—	SrFe_12_O_9_-Yb-nHA	—	—	FD and *in situ* deposition	∼300 μm	Ion release experiments
Hu et al. (2019)	Porous	DD = 90%	Ce-WH	50%	—	FD, −60 °C	∼300 μm	11.7% at 5 d (37°C ultrapure water)
Chen et al. (2018)	Porous	DD = 90%	CnHA	—	—	FD and *in situ* deposition	∼200 μm	—
Chen et al. (2021b)	Gel	PMCS	MgO	1%	—	C-CL	∼100 μm	Ion release experiments
Yu et al. (2021)	Porous	-	MnHA	50%	—	FD, −20°C	100–300 μm	—

In addition to the matrix materials, the inorganic nanoparticles added as reinforcing fillers were distinct. We classified them into two main types. One class was phosphate, including 16 studies, with most of them being nano-hydroxyapatite (nHA) or nHA incorporated with other elements (see Table 4), and six were whitlockite (WH) and phosphates of lanthanide, respectively. Another class was silicate, mainly including calcium silicate, silicate-based bioglass, and nano-clay, namely, layered silicate (Liao et al., 2019; Peng et al., 2019; Wu et al., 2019; Lee et al., 2020).

The inorganic filler proportions have important effects on the performance of scaffolds. Mostly, the nanomaterial contents of sponge-like scaffolds in this study were more than 50%, while hydrogels only contained 1% nano-fillers in most cases.

As for the physical morphology of the scaffolds, the sponge-like porous scaffold was prepared mainly by the freeze–drying technique at different temperatures in 15 studies, while composite hydrogel scaffolds were fabricated *via* covalent or non-covalent cross-linking in the others. In 14 research studies related to porous scaffolds, the surface morphology of scaffolds was obtained by scanning electron microscopy (SEM) at room temperature after thoroughly drying, followed by measurement of the pore diameter of the scaffolds using image analysis software or the through-pore size analyzer (maximum bubble pressure method) (Guo et al., 2015). Four studies regarding hydrogel scaffolds also examined and calculated the mean pore size of the scaffolds after freeze-drying. The pore size of the scaffolds was 100–300 μm in over 66% (12/18) of the studies.

Moreover, the degradation rate of scaffolds was measured *in vitro* by soaking them in 37°C PBS with or without lysozyme for several weeks in seven research studies, most ranging between 10% and 30% at 4 weeks. More details are shown in Table 5.

#### Surgery

Half of the studies set a negative control group (NCG), in which the defects of animals were left empty without any interventions or implantation, while experimental groups served as control groups mutually in the remaining of the studies with various components or different concentrations of scaffolds.

In rat models, defect areas were prepared bilaterally in the center of the parietal bones in 73% (16/22) of studies and unilaterally in 22% (5/22) of studies. The diameter of the defect areas was around 5 mm in 18 studies, while it was only 6 mm in two studies, showing that most researchers reached a consensus on the definition of the critical-sized defect in rats. Furthermore, the healing period following implantation surgery was 4–16 weeks. In most cases, it is more common to kill animals 8–12 weeks after modeling ( Table 5).

### Outcome measures

The scanning resolution of micro-CT was available in 12 studies, with no more than 20 μm in most of them (11/12). The maximum and minimum resolutions were 28 and 9 μm, respectively (Zhou et al., 2017; Yu et al., 2021). Moreover, the scanning tube voltage was reported in 11 literature studies, with no greater than 65 kV in 75% (7/11) of them. Nevertheless, none of the scanning parameters was recorded in eight studies; the threshold set for three-dimensional reconstructions and the methods for delimiting the regions of interest were not mentioned in any of the studies.

A quantitative analysis was conducted targeting 3D reconstruction from micro-CT data (see Table 6). The mBV/TV of the negative control groups in those 21 studies for rats ranged from 0.5 %–8.7%, the mean, median, and standard deviation of which were 3.5%, 2.6%, and 1%, respectively. While in the experimental groups, the mBV/TV was between 5.8% and 33.4%, with the mean, median, and standard deviation of which were 17.3%, 16.6%, and 7.0%, respectively. In addition, the BMD of the newly regenerated bone was assayed to evaluate their density and quantify the quality of mineralized tissues. However, the BMD in one research study lacked dimension (Calis et al., 2017), and another study obtained an abnormally low BMD compared with the others, possibly caused by different instruments or a wrong dimension (Wu T. et al., 2020). Moreover, studies that have calculated the Tb.Th (Zhao P. P. et al., 2019; Liao et al., 2019; Wu et al., 2019; Tang et al., 2020) or Tb.N (Zhao P. P. et al., 2019; Wu et al., 2019; Lee et al., 2020; Tang et al., 2020) are not listed.

**TABLE 6:** Surgery details and quantitative assessment results of micro-CT. NCG: negative control group; EG: experimental group.

Author (Year)	Animal	Defect location	Defect diameter	Evaluation period (months)	mBV/TV of NCG	mBMD of NCG	Scaffolds of EG	mBV/TV of EG	mBMD of EG
M. Calis et al. (2017)	Rat	Bilateral	5 mm	2	2.8	0.44	CS/B-nHA	15.4	0.44
				4	3.0	1.15		15.78	0.5
X. Ding et al. (2019a)	Rat	Right	5 mm	1	—	—	CS/DEX/Sr100-nHA	27.2	—
				2				20.7	
Y. Ji et al. (2017)	Rat	Right	5 mm	3	2.1	—	CS/PLGA/Col-nHA	15.6	—
D. Zhou et al. (2017)	Rat	Bilateral	5 mm	2	—	—	CS/WH	18.7	0.1 g cm^−3^
Lee et al. (2020)	Mouse	—	3 mm	2	—	—	PGCS/LAP	10.4	0.2 g cm^−3^
F. Liao et al. (2019)	Rat	Bilateral	5 mm	3	—	—	CS/Gd-MCS	30.8	0.22 g cm^−3^
Y. P. Guo et al. (2015)	Rat	Bilateral	6 mm	2	8.7	0.075 g cm^−3^	CS/nHA	33.4	0.38 g cm^−3^
T. W. Sun et al. (2017)	Rat	Bilateral	5 mm	3	—	—	CS/MS-nHA	13.4	—
X. Y. Peng et al. (2019)	Rat	Bilateral	5 mm	3	—	—	CS/La-MCS	28.7	0.22 g cm^−3^
H. Hu et al. (2018)	Rat	Bilateral	5 mm	3	—	—	CS/LaPO_4_	16.6	0.2 g cm^−3^
Y. Zhao et al. (2019a)	Rat	Unilateral	5 mm	3	2.3	0.01 g cm^−3^	CS/Col/Fe-nHA	7.7	0.07 g cm^−3^
Y. X. Chen et al. (2017)	Rat	Bilateral	5 mm	3	—	—	CS/MgAl-LDH	17.8	0.13 g cm^−3^
Y. Li et al. (2018a)	Rat	Unilateral	5 mm	2	2.1	—	CS/nHA	5.8	—
T. Wu et al. (2020a)	Rat	Unilateral	6 mm	1	—	0.12 mg cm^−3^	CS/QCS/Sr-nHA	—	0.23 mg cm^−3^
				2		0.11 mg cm^−3^			0.20 mg cm^−3^
				3		0.12 mg cm^−3^			0.23 mg cm^−3^
J. Wu et al. (2019)	Rat	Bilateral	5 mm	2	—	—	CS/SF/GP/Cu-BG	10.6	0.29 g cm^−3^
Chen et al. (2022)	Rat	Bilateral	5 mm	1	1.2	0.01 g cm^−3^	PMCS/MgO(5)	19	0.29 g cm^−3^
				3	0.5	0.01 g cm^−3^		8.5	0.15 g cm^−3^
Zhao et al. (2019b)	Rat	Bilateral	5 mm	3	—	—	CS/GdPO_4_	16.5	—
Tang et al. (2020)	Rat	Bilateral	-	3	—	—	CS/SrFe_12_O_9_-Yb-HA	14.2	—
Hu et al. (2019)	Rat	Bilateral	5 mm	2	—	—	CS/Ce-WH	7.8	—
Chen et al. (2018)	Rat	Bilateral	5 mm	3	—	—	CS/CnHA	11.6	0.12 g cm^−3^
Chen et al. (2021a)	Rat	Bilateral	5 mm	1	6.7	0.033 g cm^−3^	PMCS/MgO(5)	13.45	0.29 g cm^−3^
				3	2.3	0.017 g cm^−3^		7.3	0.15 g cm^−3^
Yu et al. (2021)	Rat	Bilateral	5 mm	3	2.7	0.05 g cm^−3^	CS/MnHA	8.3	0.08 g cm^−3^

Furthermore, we also accomplished a semi-quantitative analysis of images reconstructed from micro-CT data. Three studies were excluded from the analysis since the reconstructing images were not provided (Zhao P. P. et al., 2019; Liao et al., 2019; Peng et al., 2019). Regarding the other 19 studies, their scores of experimental groups ranged from 2–6, with the mean and median being 4. The highest score and the corresponding group in each study are listed and shown in Table 7.

**TABLE 7:** Semi-quantitative analysis of images derived from the 3D reconstruction of micro-CT.

Author (year)	Evaluation period (month)	NCG scores	Morphology of scaffolds	Scaffolds of EG	EG score
M. Calis et al. (2017)	4	4	Gel	CS/B-HA	6
X. Ding et al. (2019b)	1	—	Gel	CS/DEX/Sr100-nHA	3
	2				5
Y. Ji et al. (2017)	3	2	Porous	CS/PLGA/Col-HA	4
D. Zhou et al. (2017)	2	—	Porous	CS/WH	3
Lee et al. (2020)	2	—	Gel	PGCS/LAP	3
F. Liao et al. (2019)	3	—	Porous	CS/Gd-MCS	—
Y. P. Guo et al. (2015)	2	1	Porous	CS/HA	4
T. W. Sun et al. (2017)	3	—	Porous	CS/MS-HA	5
X. Y. Peng et al. (2019)	3	—	Porous	CS/La-MCS	—
H. Hu et al. (2018)	3	—	Porous	CS/LaPO_4_	4
Y. Zhao et al. (2019c)	3	3	Porous	CS/Col/Fe-HA	6
Y. X. Chen et al. (2017)	3	—	Porous	CS/MgAl-LDH	2
Y. Li et al. (2018b)	2	2	Porous	CS/HA	3
T. Wu et al. (2020b)	1	2	Porous	CS/QCS/SrHA	4
	2	3			4
	3	4			4
J. Wu et al. (2019)	2	1	Gel	CS/SF/GP/Cu-BG II	6
Chen et al. (2022)	1	2	Gel	PMCS/MgO(5)	4
	3	2			4
Zhao et al. (2019a)	3	—	Porous	CS/GdPO_4_	—
Tang et al. (2020)	3	—	Porous	CS/SrFe_12_O_9_-Yb-HA	5
Hu et al. (2019)	2	—	Porous	CS/Ce-WH	4
Chen et al. (2018)	3	—	Porous	CS/CHA	3
Chen et al. (2021b)	1	2	Gel	PMCS/MgO(5)	4
	3	2	—	—	5
Yu et al. (2021)	3	1	Porous	CS/MnHA	4

### Quality assessment

**FIGURE 3:**
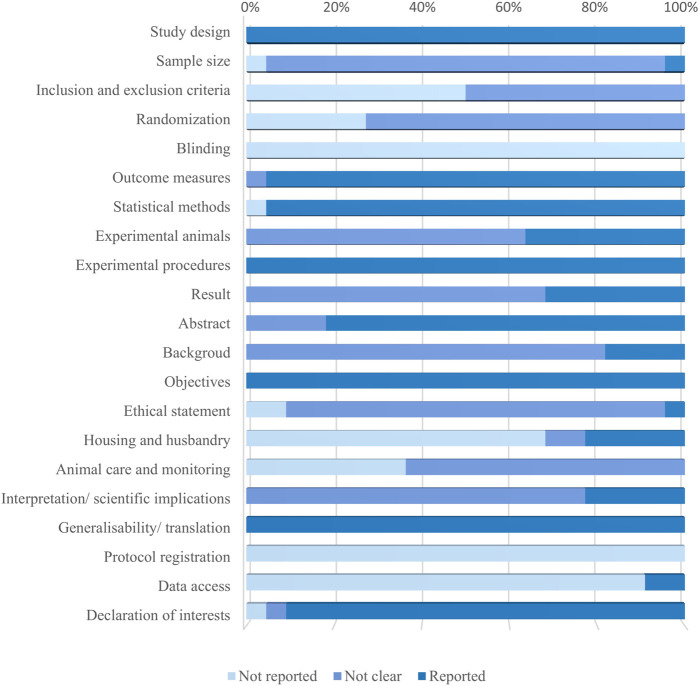
Results of the quality assessment of 22 studies applying ARRIVE 2.0 guidelines. The bar graph shows the percentage frequencies of 21 items. It is to be noted that the former 10 items belong to the essential set, whereas the latter 11 are in the recommendation set.

The percentage frequency of each item is shown in Figure 3. As for all the studies, the assessment results of “Inclusion and Exclusion criteria,” “Randomization,” and “Blinding” were “unclear” or “not reported.” Only one study was evaluated as “reported” in “Sample size.” More specifically, the methods to estimate sample size, the reasons why investigators excluded animals or data from the analysis, the approaches to randomly allocating animals in groups, and the blinding of experimental or analysis procedures were not clearly demonstrated or even mentioned.

The overall quality coefficient and assessment result of each study are shown in Table 8, ranging between 0.48 and 0.69. In total, 21 studies were classified as average quality and one as poor (Zhao Y. et al., 2019). The mean and median quality coefficients were 0.59.

**TABLE 8:** Results of the quality and general risk of bias of each study.

Author	Year	Quality coefficient	Quality	Risk of bias
M. Calis et al. (2017)	2017	0.57	Medium	Not clear
X. Ding et al. (2019a)	2019	0.60	Medium	Not clear
Y. Ji et al. (2017)	2017	0.69	Medium	Not clear
D. Zhou et al. (2017)	2017	0.67	Medium	High
Lee et al. (2020)	2020	0.57	Medium	Not clear
F. Liao et al. (2019)	2019	0.55	Medium	High
Y. P. Guo et al. (2015)	2015	0.60	Medium	Not clear
T. W. Sun et al. (2017)	2017	0.52	Medium	Not clear
X. Y. Peng et al. (2019)	2019	0.64	Medium	High
H. Hu et al. (2018)	2018	0.55	Medium	High
Y. Zhao et al. (2019b)	2019	0.48	Poor	Not clear
Y. X. Chen et al. (2017)	2017	0.62	Medium	Not clear
Y. Li et al. (2018a)	2018	0.60	Medium	High
T. Wu et al. (2020a)	2020	0.55	Medium	Not clear
J. Wu et al. (2019)	2019	0.67	Medium	High
Chen et al. (2022)	2022	0.62	Medium	High
Zhao et al. (2019c)	2019	0.60	Medium	Not clear
Tang et al. (2020)	2020	0.60	Medium	Not clear
Hu et al. (2019)	2019	0.60	Medium	Not clear
Chen et al. (2018)	2018	0.62	Medium	High
Chen et al. (2021a)	2021	0.69	Medium	Not clear
Yu et al. (2021)	2021	0.60	Medium	Not clear

### Evaluation of the risk of bias

**FIGURE 4:**
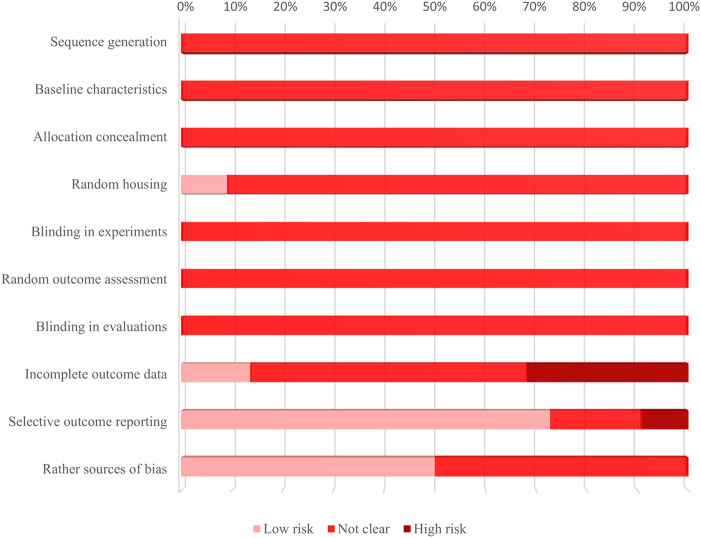
Results of the evaluation bias risk of 22 included studies applying SYRCLE’s RoB tool. The bar graph shows the percentage frequencies of 10 sources of bias. It is to be noted that the main sources of bias are “incomplete outcome data” and “selective outcome reporting.”

Figure 4 shows the percentage frequencies of each item. All of these studies have an “unclear” risk of bias in the following areas: methods for generating the randomization sequence, differences in baseline characteristics between groups, methods for allocation concealment, randomness in assessing outcomes, and blinding for outcome evaluators. Studies that were regarded to have a “high” risk of bias were identified in the areas of “Incomplete outcome data” and “Selective outcome reporting.” The former was brought on by a lack of interpretations for the causes, influences, or treatments of incomplete data, along with discrepancies in sample size between the “methods” and “results” sections, while the latter serves as a result of studies not showing the results of groups set up as negative controls.

The consequences of the overall risk of bias assessment of each study are given in Table 8. No studies achieved an overall low risk of bias. Moreover, more than one-third of the literature was evaluated to have a high risk of bias.

## Discussion

Artificial bone substitute materials have been extensively investigated for many years. Before clinical application, it is essential to conduct strict experiments *in vitro* and *in vivo* to appraise the safety and efficacy of scaffolds. The *in vivo* studies are relatively more crucial, for the outcomes of cell-aimed research are difficult to extrapolate to animals that are much more complicated.

**FIGURE 5:**
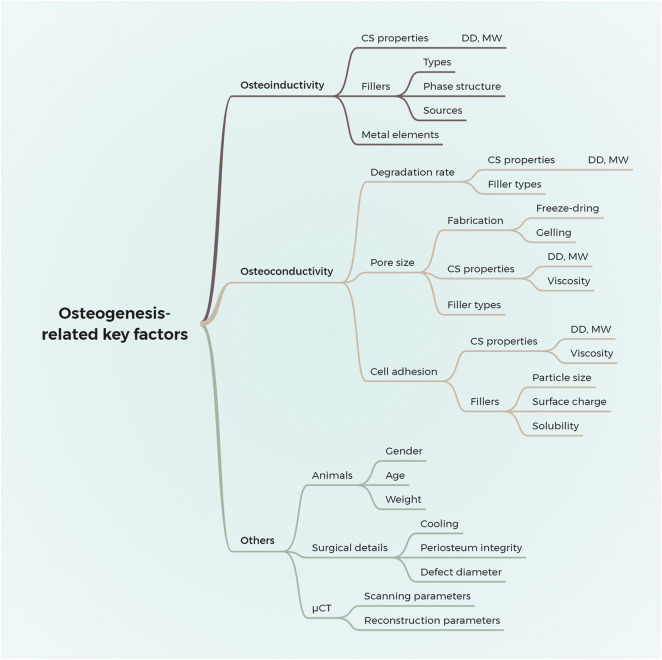
Summary of the key factors that influence the effects of chitosan/inorganic nanomaterial scaffolds on osteogenesis in calvaria CSD models. DD, the degree of deacetylation; MW, molecular weight.

We systematically reviewed the osteogenic effects of chitosan/inorganic nanomaterial scaffolds on animal calvarial bone defects. The results demonstrated that the μCT-based outcome measurements differ significantly among various studies. Furthermore, the osteogenic effects of the implanted biomaterials are affected by sophisticated factors, causing significant clinical heterogeneity, which hinders further meta-analysis or direct comparison among those scaffolds. Thus, combining the results of our systematic review, an in-depth analysis of the key factors that influence the osteogenesis of scaffolds *in vivo* is conducted as follows and given in Figure 5.

### Osteoconductivity of scaffolds

Osteoconductivity refers to the ability of the implants to be gradually substituted by the newly formed bone following the adhesion and migration of vascular endothelial cells and osteoblasts derived from host tissues into implants (Weber, 2019). In other words, the osteoconductive scaffold provides a surface and space for cells to adhere, migrate, proliferate, and differentiate. The osteoconductivity of scaffolds is determined by their morphology, structures, surface properties, and physicochemical properties.

#### Pore size

Certain space is indispensable for the life activities of cells, acting as physical structures to accommodate cells of various sizes and as channels for delivering nutrients and metabolic waste (Dziaduszewska and Zielinski, 2021). Regarding scaffolds, too-small pore sizes are not conducive for cells and tissues to grow, while excessively large pores hinder the adhesion and migration of cells (Perez and Mestres, 2016). Thus, proper pore sizes are critical for the bone regeneration properties of scaffolds. It has been reported that the pore size of trabecular bone ranges between 100 and 300 μm (Porrelli et al., 2022). Osteogenesis may be promoted by simulating the hierarchical structures of natural bones by controlling the mean pore size of composite scaffolds in these dimensions (Chen S. et al., 2021).

##### Sponge-like porous scaffolds

Manufacturing techniques for sponge-like porous scaffolds include freeze–drying, electrospinning, gas foaming, solvent casting, and 3D printing (Loh and Choong, 2013). The freeze–drying technique has been broadly applied in BTE, attributed to its process being free of toxic reagents, uncomplicated to perform, and able to make pores adjustable and controllable to a certain extent (Annabi et al., 2010). Freeze–drying is also called the ice-template method, where the water in the solution, gel, or slurry of material compounds will first be frozen under certain conditions, followed by removing the ice crystals *via* sublimation in a vacuum, and the pores will arise where the crystals are occupied. Evidently, the scaffold pore structures formed by this method are manipulated by the factors regulating the growth of ice crystals. On one hand, freezing-related factors, such as the cooling rate and final temperature, affect the growing motivation of ice crystals. The more rapid the cooling rate, the lower will be the end-set temperature, and the growing time of ice crystals is correspondingly shorter, generating smaller pores in scaffolds (Grenier et al., 2022). On the other hand, the growing resistance of ice crystals is influenced by sol- or gel-related factors such as concentrations, viscosity, pH, and chemical properties of the pre-freezing systems (Joukhdar et al., 2021), which are partially determined by the solvents and solutes.

Chitosan is a product derived from the deacetylation of chitin, composed of randomly distributed glucosamine and *N*-acetylglucosamine units linked by *β*-1,4-glycosidic bonds. The DD and molecular weight are two important performance parameters of chitosan (Takeshita et al., 2021). The number of free amino groups in a CS molecule increases with a higher DD. At acidic pH conditions, the amino groups of chitosan molecules will be protonated and positively charged, initiating inter- and intra-molecular electrostatic repulsion, making chitosan easier to disperse and dissolve in the solvent. Accordingly, the higher the DD of chitosan, the lower will be the viscosity of the chitosan solution (Dash et al., 2011; Weisspflog et al., 2021), and the easier for water molecules to migrate to the solid–liquid interface during freezing, which is conducive to the growth of ice crystals, thereby leaving larger pores in the materials after vacuum-drying (Seda Tigli et al., 2007; Joukhdar et al., 2021; Wang et al., 2021). When the DD is constant, the length of the CS molecule decreases with lower molecular weights, reducing the number of intermolecular hydrogen bonds, which promotes dispersion of CS and enlarges the size of ice crystals (Thein-Han and Misra, 2009). In addition, the content of CS in the pre-cooling mixture has a positive correlation with the pore size (Madihally and Matthew, 1999), which may be attributed to its significant effects on the viscosity of the CS solution. The Fourier transform infrared (FT-IR) demonstrated that the spectrum band, which represented the stretching vibration of the O-H bond and N-H bond, became wider and red-shifted following the higher CS content, suggesting the growth of ice crystals may be inhibited *via* the higher viscosity of the solution induced by the strengthened hydrogen-bonding effect (Murugan and Ramakrishna, 2004).

In addition to the matrix materials, fillers, namely, inorganic nanomaterials in this study, also impact the pore size of scaffolds fabricated by freeze–drying. Wu T. et al. (2020) prepared CS/quaternized CS/strontium-substituted HA (CS/QCS/SrHA) porous composite scaffolds, and then the SEM results showed that the added SrHA made the pore size smaller than for the CS/QCS scaffolds, which were influenced by the concentration of Sr Similarly, Ding et al. (2019a) also found that the Sr in HA diminished the pore diameter of the scaffolds. Li Y. et al. (2018) constructed CS/nHA/poly lactic-co-glycolic acid (PLGA) scaffolds and detected that the pore size of the scaffolds reduced with the increasing nHA content. Considering the hydroxyl groups in HA molecules, this phenomenon may be caused by the hydrogen-bonding effect mentioned previously. The previous study has proven by FT-IR that the spectrum band at about 3,400 cm^−1^ after adding nHA into the CS solution exhibits a redshift, indicating that the hydrogen bond effect is enhanced (Thein-Han and Misra, 2009; Peter et al., 2010), while further rheological detection is required to explore its effect on the solution viscosity. Likewise, Wu et al. (2019) reported that the hydrogen bond effect of the CS/silk fibroin-based scaffold was reinforced by copper-containing bioactive glass nanoparticles.

A total of 10 studies reported the freezing temperature, with six of them adopting -20 °C (Ji et al., 2017; Sun et al., 2017; Hu et al., 2018; Liao et al., 2019; Peng et al., 2019; Yu et al., 2021) and two using −80 °C (Li Y. et al., 2018; Wu T. et al., 2020). The scaffolds obtained in the former six studies had mean sizes of about 200 μm, scored as 4–5 points by our semi-quantitative evaluating system for their *in vivo* studies, while the latter two studies fabricated scaffolds with 100 μm pores and were judged as 3–4 points. Moreover, the mBV/TV of 83% of the former studies was much larger than the latter’s, with similar mBMD. Moreover, of the seven studies whose micro-CT images received 5 or 6 points, the scaffold pore size in four studies was the largest in this study, measuring up to 300 μm. These results suggest that the pore size of scaffolds plays a role in osteoconductivity.

##### Hydrogel scaffolds

Distinct from porous scaffolds with high air content, hydrogels are hydrophilic networks with high water content formed by macromolecules’ physical or covalent cross-linking. Without utilizing pore-forming techniques such as gas-foaming or particle-leaching in processing, macro-sized pores whose diameter is greater than that of a single cell do not exist in hydrogels. However, four out of the studies in this review characterized the surface morphology of hydrogels by SEM at room temperature and concluded that the mean pore sizes of these hydrogels range between 50 and 300 μm after image analysis (Ding et al., 2019a; Wu et al., 2019; Chen Y. Q. et al., 2021; Chen et al., 2022), which is debatable. Due to water-containing samples not being available for regular SEM analysis, hydrogels must be dried before scanning, which is carried out by the freeze–drying technique in most cases. As mentioned previously, freeze–drying will introduce macro-sized pores into hydrogels, destroying their original structures. Instead, to observe the real structures of hydrogels, cryo-SEM should be employed, by which water-containing samples can be scanned without dehumidification. Samples are first frozen below their glass transition temperature, at which water does not crystallize but exists in a glassy state without volume expansion and then scanned under low-temperature conditions (Bertz et al., 2013; Aston et al., 2016). Previous studies have identified by cryo-SEM that the mean pore size of the CS-based hydrogel is nanoscale (Karimi and Khodadadi, 2016).

From this point of view, the pore diameter of hydrogels without a particular pore-forming process is much smaller than that of a single cell, which makes the hydrogels poorly osteoconductive but is not supported by the *in vivo* experiments. Ding et al. (2019a) and Wu et al. (2019) implanted CS/inorganic nanomaterial hydrogels into calvarial defects of rats. After approximately a 2-month healing period, the BV/TV of experimental groups was significantly higher than that of the control groups, while the new bone regenerated along the edge of the defect without extending to the central area. However, when strontium or copper with various concentrations was incorporated into hydrogels, the defect area was almost (Ding et al., 2019a) or completely (Wu et al., 2019) covered by mineralized tissues. Their findings showed that hydrogels without macro-sized pores also have osteoconductivity in certain conditions. The mechanism of this phenomenon remains to be elucidated, which may relate to the continuous degradation of scaffolds.

#### Degradation rate

A proper degradation rate of materials that matches the tissue regeneration velocity also contributes to guiding osteogenesis (Peric Kacarevic et al., 2020). Since mineralization deposition occurs in the place where new tissue grows, if the implants are not degradable or have a degradation rate lower than the velocity of tissue regeneration, there is no space for them to grow; if much higher than that velocity, the guidance function for cells and tissues will be deprived (Zhang et al., 2014). Scaffold-related parameters are solely discussed in this review, while both host- and graft-related factors affect the balance between the degradation and regeneration rate.

As a natural linear polysaccharide, CS has been verified to be biodegradable and can be cleaved into harmless products by lysozyme *in vivo*. The degradation rate of CS depends on its molecular weight and DD. The higher the DD or molecular weight of CS, the lower the degradation rate is, with the completely deacetylated CS being unable to be enzymatically hydrolyzed *in vivo* (Yang et al., 2007).

Calcium phosphates applied for BTE include different phase structures such as HA, *β*-tricalcium phosphate (*β*-TCP), whitlockite, and calcium hydrogen phosphate (Jeong et al., 2019), while silicates mainly include calcium silicate, silicate-based bioactive glass, and nano-clay. These calcium phosphates and silicates’ degradation mechanisms *in vivo* are physical–chemical dissolution and cell-mediated biological resorption. For the physical–chemical dissolution, the mineral solubility will alter following fluctuations in the pH of body fluids in physiological conditions (Bertazzo et al., 2010; Renno et al., 2013), while a variety of cell types are involved in biological resorption, for instance, monocyte-macrophages (Xia et al., 2006), multinuclear giant cells (Lu et al., 2002), and osteoclasts (Xu et al., 2008; Long et al., 2012). Considering that the resorptive activity provoked by osteoclasts correlates with bone remodeling, osteoclast-mediated processes have been proposed to be essential for degrading grafts in osteogenesis (Sheikh et al., 2015). Furthermore, previous studies demonstrated that the degradation rate of inorganic minerals *in vivo* is determined by their crystallinity, chemical composition, and crystal structure (Stastny et al., 2019; Le Ferrec et al., 2020), and the pore size and porosity also play a part (Schaefer et al., 2011), which affects the contact area between scaffolds and the fluid environment and cells.

The degradation rate of CS-based scaffolds is significantly reduced with the addition of inorganic fillers, which could be adjusted to optimize bone regeneration by modifying the content of nanoparticles. One study in our review found that the CS/dextran hydrogels were completely degraded at 4 weeks of soaking in a simulated body fluid environment, while the degradation rate decreased to 20% under the same conditions with the incorporation of nHA. The micro-CT images showed that after the CS/dextran/nHA hydrogels were implanted in the calvarial defects, the scores were 2 points at 4 weeks and 4 points at 8 weeks, suggesting that the continuous degradation of hydrogels offered appropriate places for the newly formed bone, whose rate matched with the velocity of osteogenesis (Ding et al., 2019a). In contrast, the phytochemical-grafted CS/laponite hydrogels prepared by Lee et al. (2020) degraded 40%–50% *in vitro* at 6 weeks and were scored as 3 points 2 months after implantation, indicating that bone tissue was only deposited along the edge, and the degradation rate of scaffolds may be extremely high.

#### Cell adhesion

**FIGURE 6:**
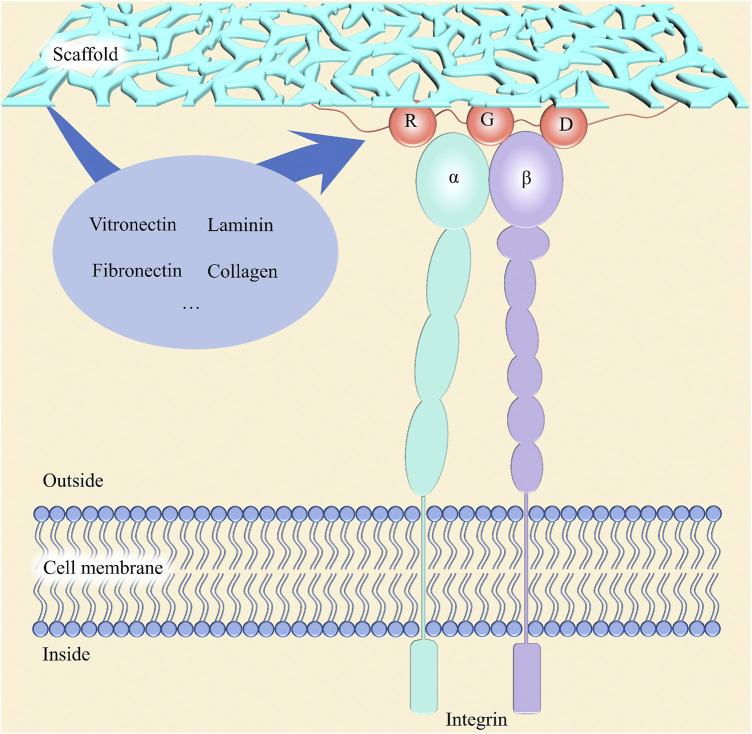
Schematic diagram for the mechanism of cell adhesion on scaffolds. Integrin, located on the cell membrane, is consisted of an *a*-subunit and a *ß*-subunit, which mediates cell adhesion to the extracellular matrix *via* binding to the RGD sequences in some proteins, such as collagen, fibronectin, vitronectin, and laminin.

The microenvironment where cells live has a critical role in cell proliferation, differentiation, and migration, which follow the step of adhesion to biomaterials. Due to the initiation of proliferation cycles of most normal human cells depending on the adhesion to a certain matrix (Jones et al., 2019), the proper adhesion ability of scaffolds is the key to guiding cells and new tissues to grow into grafts. Adhesion between cells and matrices is mediated by the integrins located on the surface of membranes (Bachmann et al., 2019). As shown in Figure 6, the domain of the integrin in the extracellular region can bind to specific ligands which contain arginine–glycine–aspartic acid (RGD) tripeptide sequences in the matrix, such as laminin, fibronectin, collagen, and vitronectin, and then the focal adhesion will form to connect cells and matrix (Ruoslahti, 1996; Bellis, 2011). Therefore, in addition to those scaffolds containing corresponding ligands, body fluid-sourced proteins adsorbed to them are indispensable for cells to attach to (Barbosa and Martins, 2017). From this perspective, surface physiochemical properties of scaffolds, such as chemical composition like groups and RGD, roughness, surface charge, and wettability, are significant parameters of adhesion ability.

Type, distribution, and the number of functional groups influence the type and number of proteins scaffolds which absorb and further affect cell adhesion through their non-covalent binding force to proteins or altering surface charge and wettability (Arima and Iwata, 2007; Yuan et al., 2011; Lieder et al., 2012). The CS deacetylated from chitin contains at least two hydroxyl groups in each fundamental unit of its molecular chain, while the number of amino groups positively correlates to the DD of the CS. Under the physiological conditions, the amino groups of CS will be protonated and confer a positive charge on it, producing a weak electrostatic attraction between CS and normal human cells, the surfaces of which are negatively charged during the resting state (Metwally and Stachewicz, 2019), whereas cells could not directly adhere to the CS molecule due to CS’s lack of structure binding to the integrin. Moreover, numerous hydrophilic hydroxyl and amino groups of CS endow the scaffolds with excellent wettability, while moderate wettability, which could be modulated by utilizing CS with different DD, is optimal for cell adhesion (Arima and Iwata, 2007).

Various inorganic nanoparticles may directly or indirectly regulate cell adhesion by altering scaffold roughness, surface charge, and surrounding ion concentrations. Previous studies have revealed that nano-scaled surface roughness affects cell adhesion (Nguyen et al., 2016; Rial et al., 2022). Webster et al. (2000) reported that the smaller the size of HA particles, the rougher will be their surface, with a significant effect on the adsorption amounts of diverse proteins. More specifically, compared to 179 nm-sized HA particles, the 67 nm-sized particles have fewer adsorptions of albumin and laminin, whereas denatured collagen and vitronectin adsorption amounts are significantly higher. In further co-culture experiments, the adhesion ability of HA to rat osteoblasts gradually increases, while that to rat fibroblasts increases, following the decline of particle size and surface roughness. Their findings revealed that the surface roughness or size of nanoparticles has an impact on cell adhesion by promoting the selective adsorption of proteins. Also, the surface of HA particles is negatively charged when in the solution at pH 7.4. With the particle size reduced to nanoscale, the zeta potential of HA is closer to 0, meaning less negative charge on its surface, which diminishes the electrostatic repulsion to cells that are also negatively charged on its surface (Cai et al., 2007). Additionally, owing to the bindings between integrins and ligands in the extracellular matrix depending upon calcium and magnesium ions, the affinity for integrins to ligands is influenced by calcium and magnesium ion concentrations in the extracellular environment. Hence, the contents of calcium and magnesium in inorganic materials and their dissociation ability also play a part in cell adhesion, which has been proven by earlier studies (Bertazzo et al., 2010; Schexnailder et al., 2010; Osada et al., 2019).

### Osteoinductivity of scaffolds

An ideal scaffold material for BTE can not only guide the growth of new tissues but also has the function of inducing mesenchymal stem cells of hosts to proliferate, differentiate into osteoblasts, and form new bones, namely, osteoinductivity, which is governed by the chemical nature of each component constructing the scaffolds and its metabolic products.

Chitosan has been proven to be biocompatible, biodegradable, and antibacterial with no osteoinductivity (Lee et al., 2020; Mahmoud et al., 2020) but has a certain extent of synergistic effect with the existence of other osteogenic factors (Mathews et al., 2011). Ding et al. (2019b) co-cultured polyethylene terephthalate (PET)/HA membranes, which have different compositions, with mouse pre-osteoblasts and detected the expression of osteogenesis-related genes. They found that compared with the PET/HA membranes, the expression of collagen-1 (COL-1), alkaline phosphatase (ALP), and osteocalcin (OCN) was upregulated in the CS/PET/HA groups, indicating that the addition of chitosan prompted proliferation or osteogenic differentiation of cells. Soriente et al. (2022) and Weir and Xu (2010) also demonstrated that the ALP activities of human bone marrow mesenchymal stem cells and mouse pre-osteoblasts co-cultured with calcium phosphates are enhanced by CS. Furthermore, researchers explored the potential effect of the DD and the molecular weight of CS on this synergism. No significant role of molecular weight has been found in osteogenesis *in vitro*, while the DD of CS is critical, with the higher the DD, the stronger will be the effect on strengthening osteogenesis (Suphasiriroj et al., 2009; Lieder et al., 2012; Alnufaiy et al., 2020).

In this review, the DD or molecular weight of CS was observed in nine studies. The DD of chitosan is over 85% in most of them. Li Y. et al. (2018) and Wu T. et al. (2020) utilized CS with the lowest DD, which may induce poor effects on osteogenesis. Micro-CT results show that the mBV/TV or mBMD of these two studies are also the lowest.

Aside from osteoconductivity, many calcium phosphates are also shown to be osteoinductive, though large variations have been reported in their osteoinductivity, which is affected by multiple factors, such as, in particular, the concentrations of osteogenic supplements in the medium, the types of cells, and the phase structure of calcium phosphates. As for nHA, some produced under laboratory conditions exhibit good performance in significantly enhancing the expression of osteogenesis-related genes (Sun et al., 2017; Ding et al., 2019a; Wu T. et al., 2020). In contrast, one study combined the commercial nHA with CS/PLGA and co-cultured with mouse bone marrow mesenchymal stem cells in osteogenic induction media, finding that the CS/PLGA/nHA composite scaffold has no significant effect on the activities of ALP of cells compared with the negative control group (Li Y. et al., 2018). Zhou et al. (2017) contrasted the different impacts of CS/nHA and CS/nWH on human bone marrow mesenchymal stem cells with dexamethasone and *β*-glycerophosphate and concluded that the osteoinductivity of nWH is better.

Multiple calcium silicates (Huang et al., 2015; Saravanan et al., 2015; Wu I. T. et al., 2020; Huang et al., 2020; Zhou et al., 2021) and silicate-based bioactive glass (Goel et al., 2012; Santocildes-Romero et al., 2015; Kroschwald et al., 2021; Marin et al., 2021; Rastegar Ramsheh et al., 2021) with different particle sizes, sources, and chemical compositions have outstanding osteoinductivity. Except for the two classical silicates for BTE, many types of nano-clay have been introduced to bone regeneration. According to phase structures, nano-clay, namely, layered silicate, is subdivided into montmorillonite, halloysite, laponite, sepiolite, and so on. To our knowledge, laponite and halloysite have been verified to have good osteoinductivity without any osteogenic elements such as dexamethasone, *β*-glycerophosphate, or *l* -ascorbic acid (Li T. et al., 2018; Shi et al., 2018; Zhao X. et al., 2019; Lee et al., 2020), whereas montmorillonite is proven to solely have a synergetic effect on osteogenesis in available research (Mieszawska et al., 2011; Kundu et al., 2021).

In manufacturing nano-particles, the incorporation of diverse metal elements impacts the osteoinductivity of scaffolds as well. In 14 studies, chitosan was combined with strontium (Ding et al., 2019a; Wu T. et al., 2020; Tang et al., 2020), iron (Zhao Y. et al., 2019; Tang et al., 2020), magnesium (Sun et al., 2017; Chen Y. Q. et al., 2021; Chen et al., 2022), and different lanthanides (Hu et al., 2018; Zhao P. P. et al., 2019; Hu et al., 2019; Liao et al., 2019; Peng et al., 2019; Tang et al., 2020), which significantly enhanced osteogenesis probably through the Wnt/*β*-catenin, Smad, or Akt/GSK-3 pathways and kept excellent biocompatibility simultaneously (Burghardt et al., 2015; Hu et al., 2018; Ding et al., 2019a; Zhao P. P. et al., 2019; Li et al., 2019; Zhu et al., 2019; Wu T. et al., 2020; Gizer et al., 2020; Tang et al., 2020), but the detailed mechanism remains unclear.

### Other factors

Apart from those aforementioned implant-related factors, the characteristics of animals are crucial, such as species, strain, gender, age, and body weight. For minimizing the effect of their growth potential on the osteogenic results of experiments, animals whose age corresponds to that of adult human beings are applied in most studies. Moreover, the estrogen levels of female animals fluctuate with the physiological cycle after sexual maturation, which will further impact the activity of osteoblasts and osteoclasts (Strube et al., 2009; Mehta et al., 2011; Haffner-Luntzer et al., 2021). Thus, only male animals are recommended to construct models for osteogenic research, except those that take osteoporosis into account. In this review, female rats were modeled in four studies without any interpretation, while the animal’s gender was not depicted in the other three, which may lead to a confusing conclusion.

Surgical details contribute to bone regeneration as well. Except for fibrous connective tissue, the periosteum covering the surface of the cranium has blood vessels and osteoprogenitor cells in its deep layer, which are favorable for osteogenesis (Roberts et al., 2015). In our unpublished study, after preparing 5-mm-sized defects at the center of the calvarias of 12-week-old rats, the intact periosteal flaps were sutured *in situ* without the implantation of any scaffold or application of osteogenic medicine. Subsequently, following a 2-month healing period, the defect areas were almost covered by new bone detected by micro-CT. Nonetheless, only two of the 22 included studies illustrated their operations to the periosteum (Calis et al., 2017; Chen Y. Q. et al., 2021), which reduces bias in their results. Moreover, cooling measures applied during drilling for defects can protect the surrounding cells from thermal injury, which would impede bone repair.

Additionally, as one of the primary quantitative assessment instruments for the effect of bone regeneration *in vivo*, scanning and reconstructing affect the final results. First, in scanning, different micro-CT instruments, pre-scanning calibration, and scanning parameters such as tube voltage, tube current, and exposure time determine the Hounsfield unit (HU) values, which represent the density of scanned tissues and are used to segment bones (Bouxsein et al., 2010; Kallai et al., 2011). Afterward, 3D reconstructions are conducted with thresholds that have no standards and are set by the subjective judgment of researchers but play a decisive role in the measurements of tissue volume (Bouxsein et al., 2010; Kallai et al., 2011). Despite this, none of the included literature in this systematic review clarified the reconstruction thresholds or methods, which poses a challenge to the reliability of these results.

## Limitations

Although we tried to conduct a detailed systematic review, limitations are inevitable in this study. First, we attempt to employ an image scoring system to estimate the osteoconductivity by assessing bone tissue coverage in the defect area. However, it is difficult to discriminate osteoconductivity and osteoinductivity *in vivo*, leading to a one-sided judgment to a certain extent, and the scoring system has a subjective nature. Second, some composite scaffolds constructed for sustained release systems may only have osteogenic properties with the existence of medicine, osteogenic factors, or cells, while only free scaffolds were taken into consideration in this review to investigate their inherent ability for bone formation. In addition, the underlying molecular mechanisms of how these materials affect osteogenesis have not been elucidated, which requires further research.

## Summary and perspective

In summary, this systematic review led us to identify good materials in this field and a pool of key factors during *in vivo* experiments and to provide modification directions for better bone regeneration capability of scaffolds in future research.

Moreover, due to the suboptimal results of quality assessment and risk of bias evaluation of included studies, for future research, we recommend referring to the ARRIVE 2.0 guidelines during animal experiments and reporting normatively, following the SYRCLE’s risk of bias tool to increase the credibility of the findings.

## Conclusion

This systematic review aimed to carry out an in-depth analysis of parameters affecting the osteoconductivity and osteoinductivity of various chitosan/inorganic nanomaterial composite scaffolds in calvarial bone critical-sized models. First, gender, age, and weight of animals impact their growth potential, which should be considered in the study design phase. During modeling, local cooling, the diameter of the defect area, and the integrity of the periosteum are critical points. As for the materials of the scaffolds, the DD, molecular weight, and viscosity of chitosan influence its application effect, while the source, chemical composition, stoichiometry, crystallinity, solubility, particle size, surface charge, and content of inorganic fillers, which are mainly divided into phosphates and silicates, are major variables. Moreover, the fabrication process, pore structure, degradation rate, wettability, and adsorption of proteins in the body fluids of scaffolds should be taken into account. Last but not least, scanning parameters and 3D reconstruction methods have important implications for assessment results.

In addition, ARRIVE 2.0 and SYRCLE’s tools were utilized to conduct quality assessment and RoB evaluation for the included studies. On one hand, 21 out of 22 studies have an average quality, while one hass poor quality. Further analysis revealed that the major factors affecting their quality are methods of determining sample size, inclusion and exclusion criteria for animals, animal randomization, and blinding in certain experiment steps, which have not been clearly reported or even been mentioned. On the other hand, over one-third of the literature has a high risk of bias, and none of the others is at a low risk of bias, mainly caused by follow-up and reporting bias.

## Data Availability

The raw data supporting the conclusion of this article will be made available by the authors, without undue reservation.
