# Erratum: Effect of chitosan/inorganic nanomaterial scaffolds on bone regeneration and related influencing factors in animal models: a systematic review

**DOI:** 10.3389/fbioe.2025.1562494

**Published:** 2025-02-03

**Authors:** Frontiers Production Office

**Affiliations:** Frontiers Media SA, Lausanne, Switzerland

**Keywords:** chitosan, inorganic nanomaterials, bone regeneration, animal models, calvarial bone defects

Following publication, we found a non-genuine email address had been provided for reviewer Alaa El-Din Bekhit. Following an investigation, which was conducted in accordance with Frontiers policy, we confirmed that the real Alaa El-Din Bekhit was impersonated and did not take any action on this manuscript and has therefore been removed from this article. In line with the COPE guidelines on potential peer review manipulation, we conducted a post-publication assessment that concluded that the article meets the standards for publication at Frontiers.

The publisher apologizes for this mistake.

The original version of this article has been updated.

